# A potentially fatal complication that may occur during dental treatment: “foreign body aspiration”

**DOI:** 10.11604/pamj.2015.20.36.5893

**Published:** 2015-01-14

**Authors:** Oguz Eroglu, Hatice Algan-Kaya, Figen Coskun

**Affiliations:** 1 Department of Emergency Medicine, Kirikkale University, Faculty of Medicine, Kirikkale, Turkey

**Keywords:** Dental treatment, foreign body aspiration, early bronchoscopy

## Abstract

Numerous systemic emergency situations, such as hypotension or allergic reactions, may be encountered during dental treatment. In addition, rare but life-threatening complications such as foreign body aspiration in the air passages may also be seen. Aspirated foreign bodies include teeth, implants, mechanical supports or materials used during procedures. We report two separate cases of aspiration risk developing during the course of dental treatment.

## Introduction

Foreign body aspiration is a potentially life-threatening condition requiring rapid treatment. Risk is greater in the geriatric and pediatric age groups than other age groups. Aspiration may occur in totally normal individuals if they speak or laugh with an object in their mouths. Food particles in the mouth or objects held between the lips, such as pins, may be aspirated. Turban needles are some of the most common foreign bodies aspirated in adulthood in Islamic countries. In addition, neurological diseases, psychological disorders, use of drugs that depress the nervous system or cause changes in consciousness, alcohol use, trauma and procedures performed under sedation (Dental or oral procedures) are all risky in terms of aspiration [1, 2].

## Patient and observation

### Case 1

A 51-year-old male patient presented to the emergency department with pain in the left posterior part of the tongue and a sensation of a foreign object in the throat from the dental clinic where he had been receiving canal treatment. DM was present in his history, but no other characteristic. At physical examination his general condition was good. He was conscious, lucid and cooperative, TA was 110/70 mmHg, pulse 90/min, RR 20/min and regular and SatO2 96. No foreign body was seen in the oral cavity or those parts of the oropharynx visible to the naked eye. Both hemithoraces contributed equally to respiration at pulmonary examination. There were no rales or rhonci at auscultation.

### Case 2

**Figure 1:**
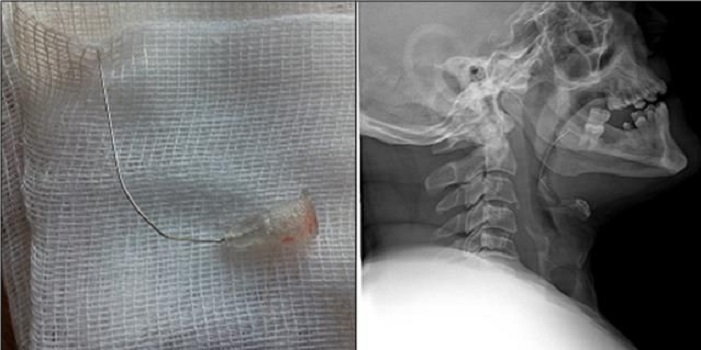
X-Ray and foreign body's of Case 1

**Figure 2:**
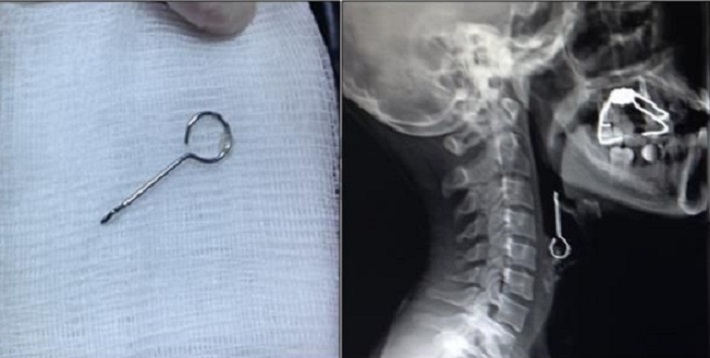
X-Ray and foreign body's of Case 2

A 23-year-old male presented with cough, shortness of breath, hoarseness and a painful sensation when speaking developing after breakage of implant material from a dental surgery he was attending for implant treatment. No significant characteristic was present in his history. At physical examination his general condition was good, and he was lucid, oriented and cooperative. TA was 120/78 mmHg, pulse 88/min, RR 20/min and regular and SatO2 98. Oropharyngeal examination revealed a dental implant between the left upper palate and the 1^st^ and 2^nd^ molars; no foreign body was observed, however. Both hemithoraces contributed equally to respiration at pulmonary examination. There were no rales or rhonci at auscultation. Air passage and pulmonary X-rays were performed in both cases. Air passage X-ray in the first case revealed a dental needle in the hypopharynx at the level of the pyriform sinus (Figure 1); in the second case a piece of implant material was observed at the level of the epiglottis (Figure 2). Ethmoid forceps accompanied by a 70-degree endoscope were used in both cases, and the foreign bodies were extracted, from the posterior of the tonsillar pole in the 1^st^ case (Figure 1) and from a left vallecula location in the 2^nd^ case (Figure 2). Control X-rays were normal, and after 4 hours observation both patients were discharged with appropriate advice with no additional problem.

## Discussion

Foreign body aspiration is one of the possible complications during dental treatment procedures. Respiratory passage aspiration or ingestion may take place in the supine or semi-lateral positions preferred by dentists during treatment [3, 4]. Local anesthesia or sedation increases the risk [5]. One rare complication is needle breakage during local anesthesia. Modern use of one-time disposable needles has reduced this possibility to a minimum. However, breakages can be seen in needles frequently sterilized with hot air. Defects associated with needle manufacture, excessive pressure during injection, bending the needle before use and sudden patient movement can all lead to needle breakage. The needle must be straight when passing through tissues, and it is incorrect to seek to overcome resistance by using force or changing the direction of the needle inside the tissue. The needle generally breaks near the intermediate part added to the injector. In the event of such a breakage, if the broken part remains inside the tissue it can easily be removed with a needle forceps. Even if the tip does not break off, a needle loosely attached to the injector can also become detached and fall into the oral cavity. The physician should try to calmly remove the object from the oral cavity without alarming the patient. If the object is completely embedded in tissue and invisible from the outside, then the position of the needle must be determined through X-ray [6, 7].

Materials that enter the gastrointestinal system after being swallowed generally cause no serious problems. They are frequently expelled without compromising peristaltis or causing complications. However, objects aspirated into the airway may give rise to potentially life-threatening conditions (Aspiration, atelectasis, perforation, inflammatory reaction, pulmonary hyperinflation, pneumomediastinum severe obstruction or death) [8, 9]. Aspiration material enters the right main bronchus more commonly than the left. This is because the right main bronchus is wider and descends in a more perpendicular manner as the continuation of the trachea. The left main bronchus is more angles and narrower. However, this difference and angling between the left and right main bronchi takes place after the age of 15. Right and left bronchus aspiration levels are very similar, therefore, in young children [1]. Imaging techniques such as direct X-ray, bronchoscopy and computerized tomography are used in the diagnostic management of foreign body aspiration. Chest X-ray is the first diagnostic technique when tracheobronchial foreign body aspiration is suspected. Foreign body aspiration cannot be excluded in patients causing clinical suspicion but with negative direct X-ray findings. Multislice CT (MSCT) should be available to avoid bronchoscopy [8, 10].

Early diagnosis and treatment are of great importance due to the severe early and late foreign-body complications that may arise. Inflammation and granulation may develop around the foreign body in delayed cases. Basic mucosal changes such as edema and/or purulent secretion may be observed in children. Tissue reaction expectation in patients treated within 24 h is 0.8%, but 20% and 100%, respectively, in cases treated between 2 and 20 days and cases treated after 30 days [11]. Rigid bronchoscopy under general anesthesia is the gold standard for diagnosis and treatment management. However, flexible bronchoscopy is now widely available and is much preferred in foreign body extraction by suitably trained physicians [12, 13]. There is no consensus on the most appropriate technique for the extraction of foreign bodies in the trachea. Some authorities report a success rate of 95% with rigid bronchoscopy, while others report a success rate of 90-92% with flexible bronchoscopy [14].

## Conclusion

We describe two separate cases of foreign bodies swallowed during dental treatment and attaching to the proximal part of the air passage. Rigid or flexible bronchoscopy equipment should be available in emergency departments for the rapid diagnosis and treatment of foreign bodies aspirated in the air passage, and personnel trained in their use must also be available.
